# An One-step Triple Enhanced weakly supervised semantic segmentation using image-level labels

**DOI:** 10.1371/journal.pone.0309126

**Published:** 2024-10-21

**Authors:** Longjie Quan, Dandan Huang, Zhi Liu, Kai Gao, Xiaohong Mi

**Affiliations:** 1 School of Electronics and Information Engineering, Changchun University of Science and Technology, Changchun, People’s Republic of China; 2 National and Local Joint Engineering Research Center of Space Photoelectric Technology, Changchun University of Science and Technology, Changchun, People’s Republic of China; 3 School of Business, Henan University of Science and Technology, Luoyang, People’s Republic of China; South China University of Technology, CHINA

## Abstract

Weakly supervised semantic segmentation, based on image-level labels, abandons the pixel-level labels relied upon by traditional semantic segmentation algorithms. It only utilizes images as supervision information, thereby reducing the time cost and human resources required for marking pixel data. The prevailing approach in weakly supervised segmentation involves two-step method, introducing an additional network and numerous parameters, thereby complicating the model structure. Furthermore, image-level labels typically furnishes only category information for the entire image, lacking specific location details and accurate target boundaries during model training. We propose an innovative One-Step Triple Enhanced weakly supervised semantic segmentation network(OSTE). OSTE streamlines the model structure, which can accomplish both pseudo-labels generation and semantic segmentation tasks in just one step. Furthermore, we augment the weakly supervised semantic segmentation network in three key aspects based on the class activation map construction method, thereby enhancing segmentation accuracy: Firstly, by integrating local information from the activation map with the image, we can enhance the network’s localization and expansion capabilities to obtain more accurate and rich location information. Then, we refine the seed areas of the class activation map by exploiting the correlation between multi-level feature. Finally, we incorporate conditional random field theory to generate pseudo-labels with higher confidence and richer boundary information. In comparison to the prevailing two-step weakly supervised semantic segmentation schemes, the segmentation network proposed in this paper achieves a more competitive mean Intersection over Union (mIoU) score of 58.47% on Pascal VOC. Additionally, it enhances the mIoU score by at least 5.03% when compared to existing end-to-end schemes.

## 1. Introduction

Semantic segmentation is a crucial technology in computer vision, dividing images into segments with different semantics, identifying the semantic category of each segment, and ultimately achieving pixel-by-pixel labeling for a segmented image.

However, mainstream semantic segmentation algorithms often demand a substantial amount of labeled data, requiring painstaking and costly pixel-by-pixel annotation. To alleviate this burden, researchers have turned their attention to algorithms that efficiently construct labels, with weakly supervised semantic segmentation emerging as a notable approach. Instead of relying on traditional pixel-level labeled data, weakly supervised semantic segmentation employs coarser yet more concise labeled data for model training. There are four common forms of weakly supervised semantic segmentation based on different labeling methods.

**Fig 1:**
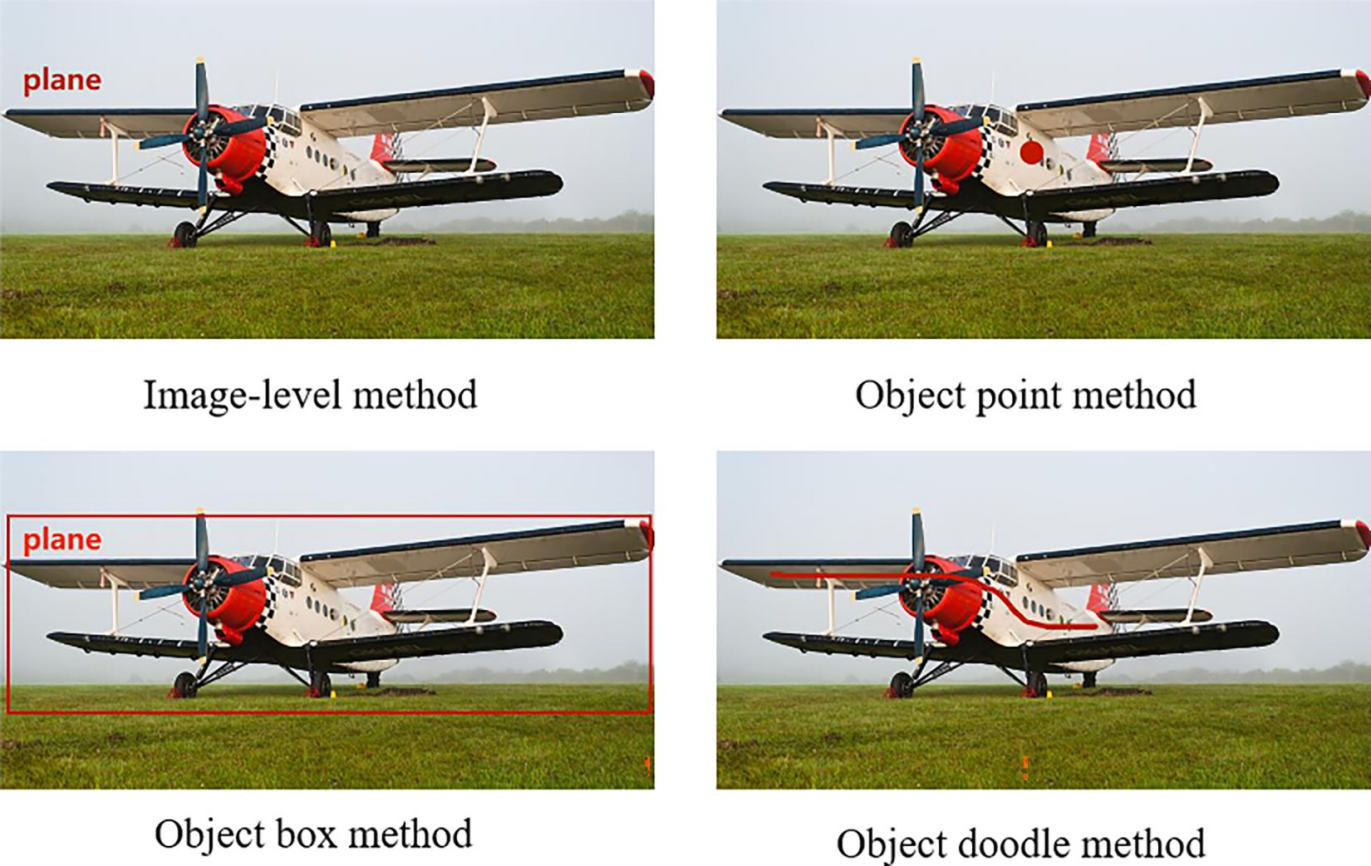
Labeling forms for weakly supervised semantic segmentation. The accuracy of semantic segmentation for these four methods generally increases with the amount of positional information they provide. However, the complexity of the models also increases accordingly.

Image-level method: This method involves labeling only the category to which the relevant object in the image belongs, such as the label shown in the upper right corner of Fig 1. It is the simplest form of annotation [1] and does not provide any location information about the object.

Point-level method: This method involves labeling only a point on the object along with its corresponding category [2], as shown by the red dot on the airplane. This provides unique location information for the object.

Bounding box-level method: This method involves labeling the rectangular box where each object is located in the image along with its corresponding category [3]. This provides rough positional information about the object but no detailed boundary information.

Scribe-level method: This method involves drawing a line on each object and labeling the corresponding category [4], such as the red curve on an airplane. This usually provides relatively detailed positional and boundary information about the target.

Despite the relatively low segmentation accuracy of image-level method, they entail minimal effort in data labeling and utilize the simplest models. This inherent simplicity presents substantial potential for improvement and significant research value. In this study, we will primarily concentrate on semantic segmentation employing image-level method.

Current segmentation methods using image-level label as supervised information are categorized into two-step and one-step methods. Most studies have employed two-step methods [5, 6], which exhibit higher segmentation performance and are closer to fully supervised segmentation methods. Generally, this method initially utilizes image-level labels as supervised information to generate high-quality pixel-level pseudo-labels with a classifier. Subsequently, these pseudo-labels replace the true-value labels are fed into, for example, a fully convolutional network to train the semantic segmentation model. However, to generate high-quality pseudo-labels, the two-step approach incorporates a multi-instance learning mechanism or saliency map, introducing additional foreground-background cues. This introduces a new network into the already complex two-step training, making the two-step method structurally large and challenging to implement. In contrast, one-step methods [7] typically establish an end-to-end framework. These methods possess a simpler structure and can save training resources for efficient and rapid learning. However, one-step methods do not employ constrained optimization strategies to enhance learning. They focus solely on training speed, sometimes neglecting segmentation accuracy, and their semantic segmentation results often fall short of those achieved by two-step methods.

In fully-supervised semantic segmentation, labels serve the dual purpose of conveying category information and specifying the precise location of each pixel. This pixel-level labeling is pivotal for training and evaluating semantic segmentation models, as the model must adeptly associate each pixel with the correct category while retaining spatial information to achieve accurate segmentation of distinct objects or regions in the image. When image-level label is employed as the initial supervisory information, it provides only semantic category information for objects in the overall image, lacking specific location details. Only by acquiring sufficiently precise and abundant location information can the final generated pseudo-label closely approximate the actual label, thereby enhancing the segmentation effect.

To balance model simplicity with higher semantic segmentation accuracy, we present the One-step Triple Enhanced (OSTE) network. OSTE requires only one step to accomplish both the generation of pseudo-labels and the training of segmentation networks in a weakly supervised task. OSTE initially partitions the input image into smaller localized images, facilitating the extraction of location information for localized features of the target, thereby enhancing the activation-like map’s localization capability. Subsequently, it integrates correlations between multi-scale and multi-level feature pixels to refine the seed region. It then employs a conditional random field and threshold selection method to identify pixels with higher confidence, enhancing annotation quality. The pseudo-labels encompass non-discriminative regions of the object, with detailed and smooth boundary parts that closely resemble manually labeled annotations. The pseudo-labels encompass non-discriminative regions of the object, with detailed and smooth boundary parts that closely resemble manually labeled annotations. Additionally, this paper introduces the average absolute error and Gaussian kernel energy loss functions alongside the cross-entropy loss function to optimize the training process. In summary, our contribution is as follows:

·We explore a simple and efficient network structure based on the one-step method(OSTE), which can perform both pseudo-label generation and semantic segmentation tasks end-to-end.

·We propose the Location Expansion Module, which enhances the model’s ability to extract location information from image-level labels by splitting and merging images in varying quantities.

·We propose the Multilevel activation fusion module, which refines the seed area of the class activation map. And combined with the Foreground-background Enhanced Module, screening high-confidence foreground and background pixels to get higher quality pseudo-labels.

·The OSTE achieves an mIoU score of 58.47% on the Pascal VOC 2012 dataset, demonstrating segmentation performance comparable to the two-step method and a distinct advantage over existing end-to-end solutions.

## 2. Related work

### 2.1 Image area localization based on weakly supervision

In weakly supervised semantic segmentation, two common methods for target localization are Semantic Localization (SeLo) and Class Activation Mapping (CAM).

In recent years, there has been progress in the development of SeLo, a multi-modal, advanced weakly supervised model that leverages additional information such as text and bounding boxes to localize relevant targets within remote sensing images. Yuan et al. [8] provide a comprehensive quantitative evaluation of SeLo’s performance using various metrics. Yu et al. [9] employ multilevel likelihood expansion techniques to expedite cross-module similarity computation within SeLo. They effectively utilize low-frequency semantic information while leveraging high-frequency semantic cues to enhance SeLo’s accuracy.

**Fig 2:**
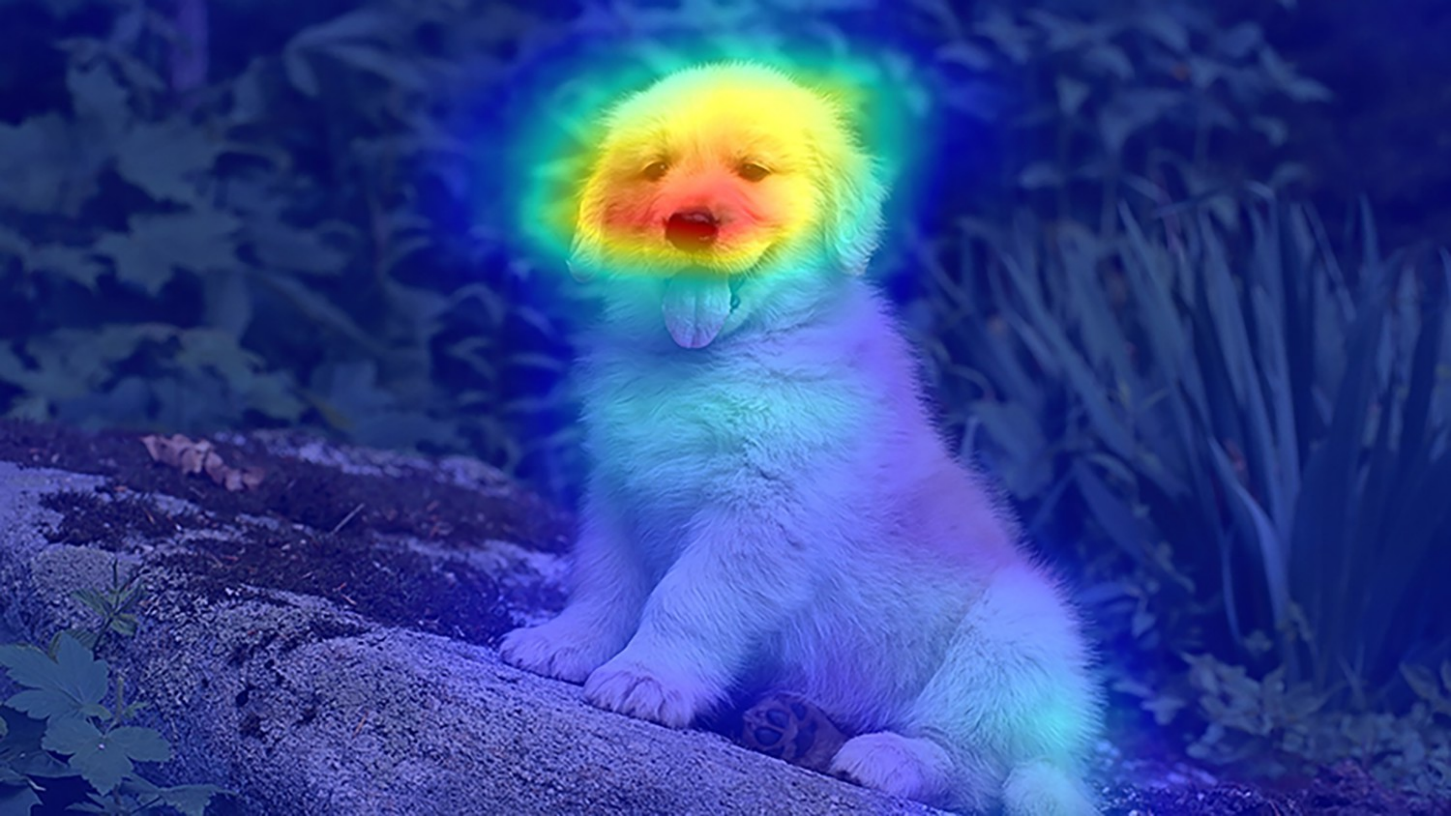
CAM of dog. CAM usually focuses on the most salient features. Therefore, only the head of the dog is highlighted in the image; the rest of it cannot be the defining feature of the dog.

CAM [10] plays a crucial role in making deep learning decisions interpretable and transferable. It is widely applied in weakly supervised semantic segmentation tasks for non-remote sensing images. CAM enhances target localization by designing a weight generation method and classifier structure corresponding to different feature maps. Grad-CAM [11] and Grad-CAM++ [12] utilize class-specific gradients to compute weights for each feature map. Score-CAM [13] instead utilizes feed-forward scores to generate weights. Puzzle-CAM [14] improves CAM by constructing a regularized loss derived from both the split CAM and the original CAM, thereby enhancing the CAM’s quality. Layer-CAM [15] breaks away from depending on the final convolutional layer to generate CAM. It can extract reliable class activation maps from different layers of the CNN while generating weights. But CAM concentrates on the most discriminative features of the object, it generates a small seed region, which affects the accuracy of pseudo-label. As illustrated in Fig 2, the highlighted region in the CAM for the dog focuses solely on the head. So we propose the Location Expansion Module, which captures richer target features in each block sequentially by splitting and combining the images.

### 2.2 Refinement of seed area

The seed area is shaped based on the features captured by the CAM, and then pseudo-labels are generated by propagating the semantic information from the seed area to the entire image. However, the initial seed area suffers from a large number of misclassification problems and the target lack detail information. To address this limitation, there is a need for further expansion of the seed region to acquire more accurate pseudo-labels.

The first category of refinement methods involves propagating object regions from the seed region to semantically similar pixels in the vicinity. This method employs a random-walk algorithm on a transformation matrix, where each element represents a similarity score [16–18]. The second method leverages saliency maps to extract background cues and integrates them with object cues [19–21]. The third method employs post-processing techniques to enhance the quality of the CAM and indirectly improve the initial seed regions it generates. Adv-CAM [22] employs gradients with respect to the input image to perturb the image, iteratively finding newly activated pixels. SEAM [23] enhances the consistency of the CAM extracted from different transformations of the image. Wei et al [24] proposed iteratively erased discriminative regions that could yield more seed regions. RIB [25] suggested retraining the multi label classification model without the last activation function. In contrast, Re-CAM [26] does not remove any activation function but adds a loss based on softmax activation. Zhou et al. [27] pioneered the conversion of classification supervision from images to pixels, imposing robust constraints on the classifier. They also introduced dynamic multi-scale fusion techniques to improve segmentation accuracy, particularly in multi-category complex scenes. CISM [28] adopts cross-image semantic mining strategies to augment feature representation, enabling the extraction of comprehensive regional and semantic information pertaining to the target. In this paper we propose Multilevel Activation Fusion module, which enables CAM to capture multi-level and multi-scale global contextual information and enhance CAM’s attention at object boundaries and details based on long-range pixel correlation. The refined CAM suppresses misclassification on targets and further generates seed area with more boundary detail information. Finally, higher quality pseudo-labels are generated after conditional random field and threshold selection methods.

## 3. Primary methodology

### 3.1 Overview

**Fig 3:**
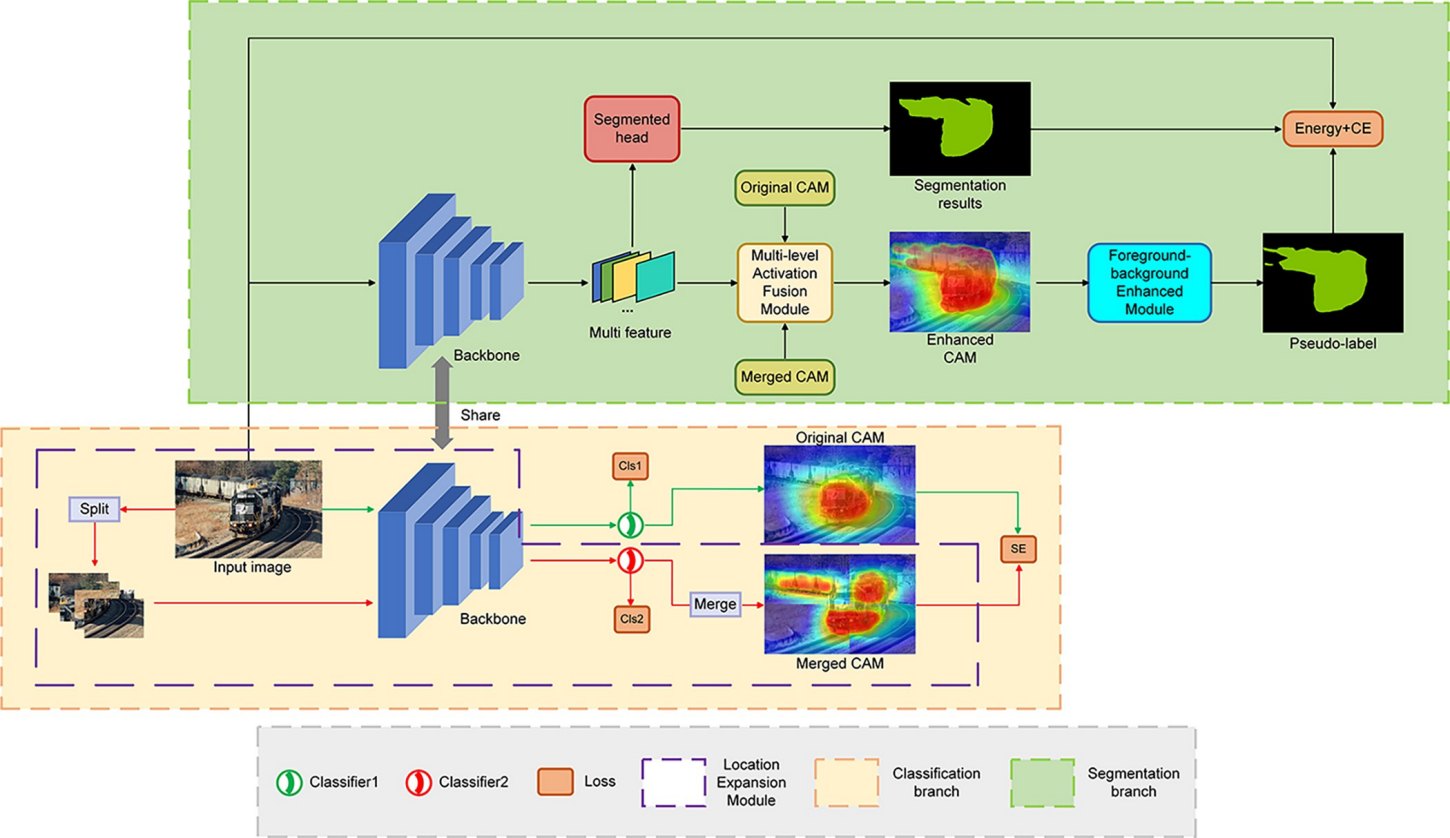
Overall framework. Its structure consists of two branches: the classification branch is utilized to generate merged CAMs, while the segmentation branch employs optimized enhanced CAMs to produce pseudo-labels, which are then utilized as supervision for training the segmentation network.

In this section, we provide a comprehensive explanation of the principles underlying the proposed OSTE network, elucidating how the network generates a more comprehensive and detailed target CAM based on image-level labels and subsequently refines the pseudo-labels used in training semantic segmentation networks. The overarching framework of the OSTE network is depicted in Fig 3: the image undergoes extraction from the initial backbone network and subsequently traverses a classifier to yield the original CAM. Employing the Location Expansion Module, the image is divided into several non-overlapping chunks through iterative operations, resulting in the merged CAM. The two CAMs are fused with input image features via the Multilevel Activation Fusion Module, yielding the augmented CAM. Further augmentation of the augmented CAM is performed using the Foreground-background Enhanced Module to derive detailed pseudo-labels. These detailed pseudo-labels then serve as the supervisory information for the segmentation branch during training.

### 3.2 Getting location information

In this paper, we employ CAM to achieve discriminative target localization. This is accomplished through the global average pooling layer and the weights of the classifiers, extracting location information from image-level labels. The obtained location information is then used to generate seed areas for each training image, facilitating subsequent pseudo-label generation tasks. It benefits from the advantages of residual networks(ResNet) in structural complexity, computational efficiency, training stability, and good performance on medium-scale datasets. We choose it as the backbone network of two branches in OSTE to extract features. It has been pre-trained in the Imagenet dataset.

#### 3.2.1 Generation of CAM

The input image undergoes processing through the backbone network to derive the feature map. In this study, modifications are made to the structure of the conventional feature extraction network, as outlined below:Firstly, the pooling layer following each convolutional layer is removed to enhance the resolution of the feature map. Eliminating the translational invariance introduced by the pooling layer helps retain more spatial positional information. This adjustment is aimed at fortifying the CAM’s sensitivity to the object’s position and, consequently, improving object localization capabilities. Secondly, the convolutions in the last three residual cells of the residual network are substituted with three layers of dilated convolutions featuring different dilation rates. This alteration preserves the image at a higher resolution and expands the receptive field without an increase in computational effort [29]. Convolving the feature map with varying rates of dilation enables the propagation of feature information from the highly responsive part of the target object to neighboring regions at multiple scales. This process enlarges the seed region, thereby enhancing the quality of the CAM generated by the classifier.

**Fig 4:**
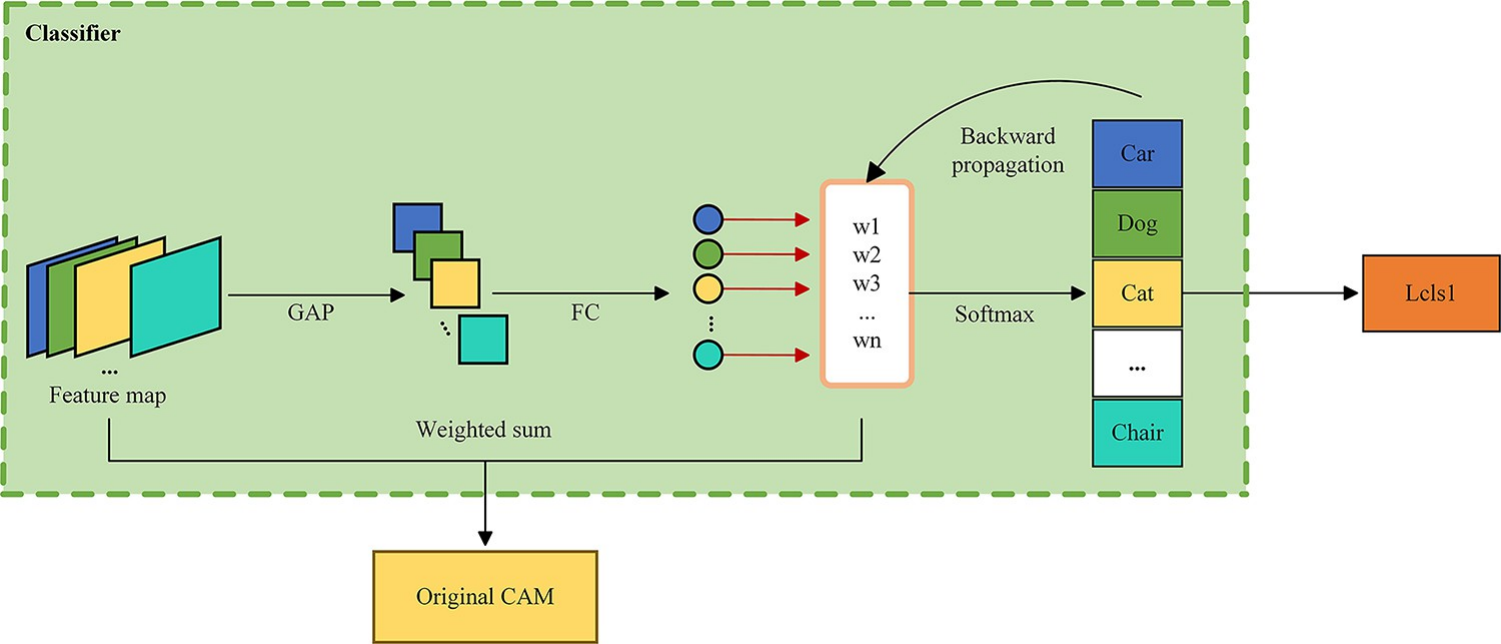
Structure of classifier. It is a simple classification network that utilizes extracted deep-level features for global average pooling. Through backward propagation, it obtains the weights w for each category. Finally, these weights are multiplied with the feature maps and summed to yield the original CAM.

The classifier illustrated in Fig 4 for generating the CAM undergoes a multi-step process. Initially, the pre-trained image classifier forwards input images, extracting feature representations rich in semantic information from the final layer. Subsequently, a global average pooling operation is applied to obtain weights for each channel, reflecting their contribution to classification. Higher weights signify greater importance. These weights are then multiplied with corresponding feature maps, emphasizing important regions (seed areas). A fusion operation combines the weighted feature maps of each channel, yielding the final CAM. This comprehensive process enhances object localization and underscores crucial features for accurate classification.

The expression for the CAM for class *k* in input image *I* can be formulated as follows:

$$M^{k}=Trans(\sum_{ch=1}^{C}F_{final}(I)\cdot W_{ch}^{k}),$$ (1)

Where *C* represents the number of channels in the feature map, *F*_*final*_(*I*) denotes the feature map obtained by convolving the last layer of the backbone network with the input image, $W_{ch}^{k}$ symbolizes the weights associated with each channel in the fully connected layer when the classification category is *k*, and *Trans*(∙) signifies the operation used to resize the resulting CAM to match the dimensions of the input image *I*.

As various objects exhibit distinct features across different scales, leveraging multi-scale raw images enables the model to capture a more extensive range of feature information. Given that the quality of CAM relies on the model’s capability to comprehend image features, this paper introduces multi-scale CAM fusion to enhance CAM quality, particularly for small-sized objects, by capturing finer details. The expression for multi-scale CAM fusion is presented below:

$$M_{MS}^{k}=\frac{1}{N}\sum_{i=1}^{N}S_{i}(M^{k}),$$ (2)

Where *S*_*i*_ represents the scaling factor, and $M_{MS}^{k}$ is the multiscale fusion CAM for class *k* of image *I*. The utilization of multi-scale CAM yields more precise object localization and detailed characterization compared to the single scale (*S*_*i*_ = 1) class of activation maps *M*^*k*^. The multiscale CAM, denoted as B, encapsulates the multiscale fusion CAM for class *k* of image *I*.

### 3.3 Localization enhancement for CAM

In ordinary CAMs, localization is limited to the most discriminative parts of an object, resulting in incomplete seed regions for generating detailed pseudo-labels. Consequently, the final trained network may struggle to segment complete objects. The core concept of iterative erasure of CAMs [24] involves erasing the corresponding part of the original image whenever a CAM localizes a discriminative object part. This process compels the classifier to search for additional discriminative regions, ultimately generating CAMs that identify more discriminative areas. In this paper, we introduce a Location Expansion Module based on this idea.

**Fig 5:**
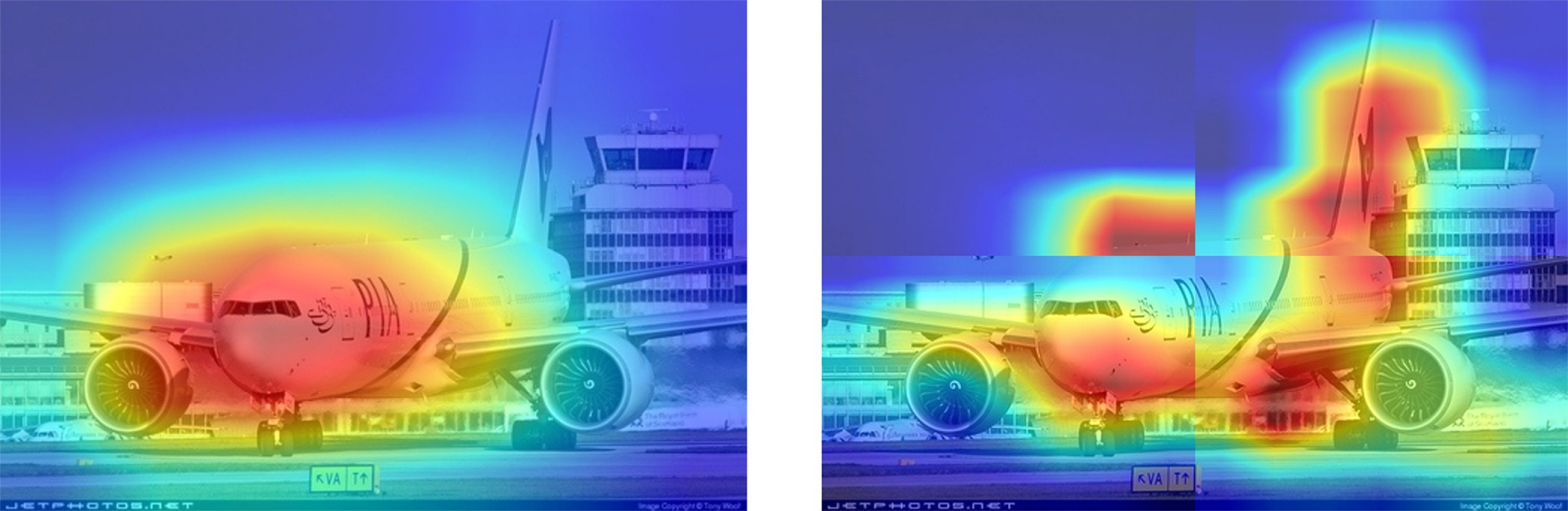
Original CAM and location extended CAM on Image Net dataset. The CAM on the left only covers features of the fuselage, whereas the CAMs split on the right label the aircraft’s subtle features such as wings and propellers.

The impact of the Location Expansion Module is illustrated in Fig 5: the higher color temperature indicates more significant features of the location identified as an aircraft, reflecting a stronger ability to locate the aircraft. Ideally, the red color should accurately and completely cover the entire fuselage. The original CAM on the left, however, only highlights the nose of the airplane, leaving the right propeller and tail, less discriminative features, untouched by the red color. Although the CAM with the Location Expansion Module is not perfect, it can identify and localize weaker parts of the image compared to the original CAM. After different scales of splitting and CAM fusion, the coverage of the red color becomes more complete.

**Fig 6:**
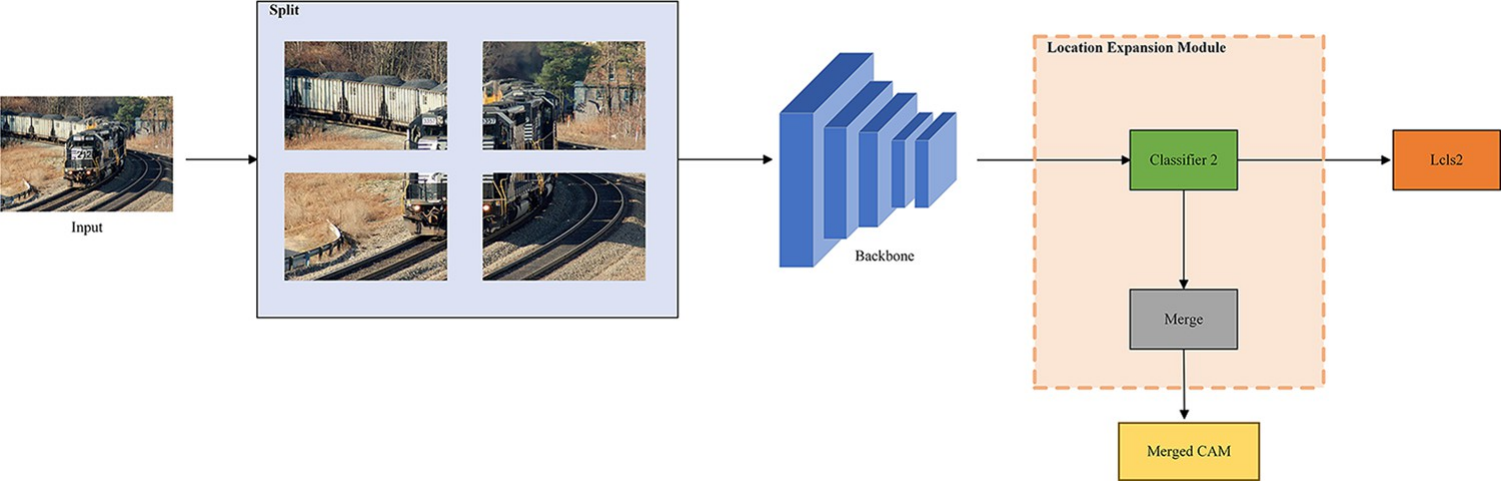
Location expansion module. It divides the image into several sections, forcing the network to identify weaker discriminative features of objects. Finally, it merges the resulting CAMs which extracted more object features and positional information.

In Fig 6, the Location Expansion Module demonstrates the splitting of the image into halves of the original size. The module, crafted in this paper, initiates by dividing the input image *I* with dimensions HxW into non-overlapping image blocks. These blocks have widths and heights of 1/2 and 1/4 of the original image size, respectively, denoted as:

$$I_{1/2}^{\prime }=\{\begin{array}{cc} I_{\left(1,1\right)}^{\prime } & I_{\left(1,2\right)}^{\prime }\\ I_{\left(2,1\right)}^{\prime } & I_{\left(2,2\right)}^{\prime } \end{array}\},$$ (3)

$$I_{1/4}^{\prime \prime }=\{\begin{array}{ccc} I_{\left(1,1\right)}^{\prime \prime } & \cdots & I_{\left(1,4\right)}^{\prime \prime }\\ \vdots & \ddots & \vdots \\ I_{\left(4,1\right)}^{\prime \prime } & \cdots & I_{\left(4,4\right)}^{\prime \prime } \end{array}\},$$ (4)

Following this, all the image blocks for $I_{1/2}^{\prime }$ and $I_{1/4}^{\prime }$ are input into the classifier, resulting in 4 and 16 split small-size CAMs, respectively. These splited CAMs are then amalgamated to form merged CAMs of the original image sizes, denoted as $M_{1/2}^{k}$ and $M_{1/4}^{k}$. The two are added to yield the merged CAMs after localization of the expansion module, expressed as follows:

$$M_{merge}^{k}=Add(M_{1/2}^{k},M_{1/4}^{k}),$$ (5)

The rationale behind the splitting is to compel the classifier to glean information from weaker discriminative regions in the local features of image blocks that lack such features. This facilitates the extension of the seed region by each small CAM block, covering a more comprehensive semantic region of the target class. The amalgamation of multiple CAMs subsequently enhances the overall completeness of the seed region. The specific choice of splitting the image into halves and quarters of the original size will be elaborated upon in next section.

### 3.4 Post-processing for CAM

While the introduction of the Location Expansion Module compels the classifier to provide more comprehensive coverage of the discriminative object region, there is a risk of misclassification in encountering certain image blocks without the target category, potentially identifying the background as the seed region. Furthermore, utilizing a standard convolutional layer for seed region extension may result in a segmentation outcome with an insufficiently continuous and smooth boundary, lacking detailed positional accuracy.

**Fig 7:**
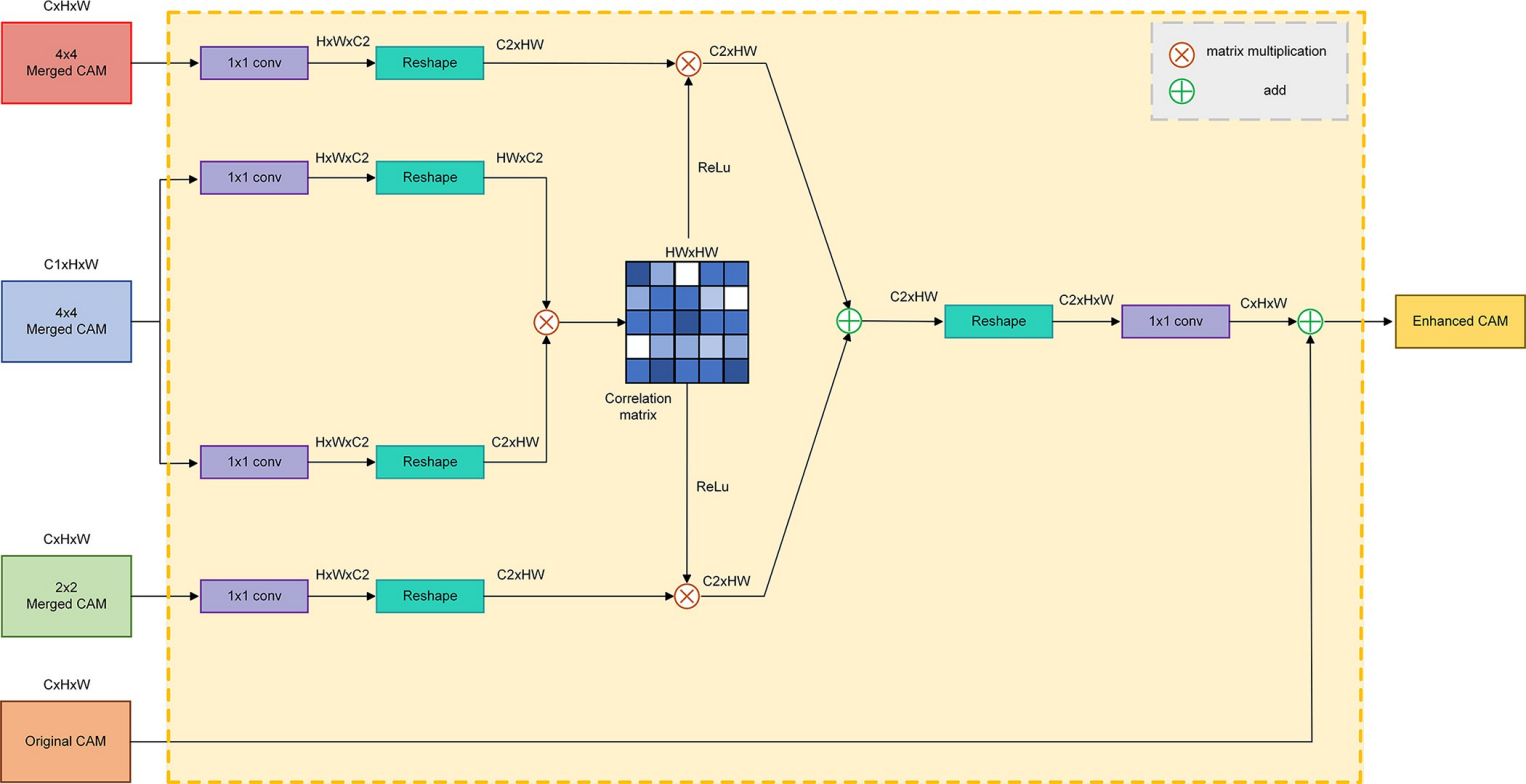
Multilevel activation fusion module. It utilizes merged CAMs to compute pixel-wise correlations and then multiplies and merges them with CAMs from different combinations. Ultimately, these are superimposed onto the original CAM, resulting in an enhanced CAM with richer boundary information.

To address these challenges, we introduce a multilevel activation fusion module based on a nonlocal self-attention mechanism. It is an arithmetically simple module for CAM post-processing to refine the seed area. This module captures global contextual information from shallow to deep multi-scale feature maps, pixel by pixel, computing long-range pixel similarity. This aids the network in distinguishing semantic categories at the pixel level, thereby enhancing boundary and detail predictions. The module involves two 1x1 convolutions and transposition operations on feature maps from the backbone network. The results are multiplied, generating a matrix of scores representing pixel correlations. After ReLu activation to remove scores less than 0, similar operations are applied to merged CAMs of different block sizes obtained by the Location Expansion Module. These are then weighted, fused with pixel correlation scores, and combined with the original CAM to produce the enhanced CAM. The structural diagram of the multilevel activation fusion module is depicted in Fig 7.

The fundamental concept of the self-attention mechanism involves calculating correlations between each element and others in the input sequence, then utilizing these correlations to assign weighted values to each element, producing a weighted representation. The non-local self-attention mechanism extends this basic idea to capture distant dependencies more effectively. The expression for the self-attention mechanism is as follows:

$$y_{i}=\frac{1}{C(x_{i})}\sum_{\forall j}f(x_{i},x_{j})\cdot g(x_{j})+x_{i},$$ (6)

Where *x* and *y* denote the input features and output features respectively, *i* and *j* denote the indexes of different spatial locations on the feature map, *C*(*x*_*i*_) = ∑_∀*j*_*f*(*x*_*i*_,*x*_*j*_) denotes the normalization function for the output features, the feature similarity of the indexes of different locations is computed using the function *f*(*x*_*i*_,*x*_*j*_), and the function *g*(*x*_*i*_) is employed to better extract the input features.Our Multilevel Activation Fusion structure represents a partial modification of the core algorithm inspired by the non-local self-attention theory. Initially, we employ cosine similarity to quantify the degree of feature similarity between distinct locations within the feature map:

$$f\left(x_{i},x_{j}\right)=\frac{\theta {\left(x_{i}\right)}^{T}\cdot \phi (x_{j})}{\left\|\theta (x_{i})\|\cdot \|\phi (x_{j})\right\|}$$ (7)

Where *θ* and *φ* denote convolutional layers of size 1x1.

The final complete expression is as follows:

MiCAM=1C(xi)∑∀j{ReLU[f(xi,xj)]⋅∑n=1/2N={1/2,1/4}g(Mjn)}+MiMS, (8)

Where $M_{i}^{CAM}$ denotes the enhanced CAM with more detailed seed regions after the multilevel activation fusion module, *x* denotes the fused feature map of shallow and deep features generated by the image after the residual network, *g* denotes the convolutional layer of size 1x1, $M_{j}^{n}$ denotes the two CAMs obtained after splitting into 4 and 16 blocks by the Location Expansion Module in earlier section, and $M_{i}^{MS}$ denotes the CAM generated in preceding section.

We replace softmax with the ReLU activation function to suppress negative values in the similarity matrix to zero, indicating that only pixels with similarity scores greater than zero are considered correlated with each other, while ignoring correlations between pixels with similarity scores less than zero. This introduction of nonlinearity aids the model in better capturing complex relationships between pixels.

### 3.5 Generation of pseudo-labels

A common method for generating pseudo-labels is thresholding. Initially, an appropriate threshold is chosen to filter high activation values in the seed regions of the CAM. The CAM is then segmented into regions based on the set threshold, where pixels above the threshold are retained, and those below are marked as 0. Subsequently, a normalization operation is applied, emphasizing regions with high activation values. Finally, based on the thresholding results, the candidate regions associated with the pseudo-labels are obtained and labeled with their corresponding semantic categories.

In this paper, our Foreground-background Enhanced Module is designed, incorporating thresholding and Conditional Random Field (CRF). Following the refinement of the seed region in earlier discussion, the CAM of each category undergoes a linear normalization operation in the channel dimension to obtain the foreground classification probability for each pixel:

$$S_{fg}^{k}(i)=\frac{M_{CAM}^{k}(i)-\min(M_{CAM}^{k}(i))}{\max(M_{CAM}^{k}(i))-\min(M_{CAM}^{k}(i))},$$ (9)

Where *i* represents the pixel position index, $\min(M_{CAM}^{k})$ denotes the minimum value in the Class *k* CAM, $\max(M_{CAM}^{k})$ denotes the maximum value in the Class *k* CAM, and $S_{fg}^{k}$ is the foreground probability score.

The expression for calculating the background score is as follows [17]:

$$S_{bg}(i)=1-\max{\left(S_{fg}^{k}(i)\right)}^{\gamma },\gamma >1,$$ (10)

Where *γ* is the attenuation rate that suppresses the background, and *S*_*bg*_ is denoted as the background probability score, which is obtained by subtracting the highest score at the same position *i* in the *k* class of foreground scores by 1 after obtaining the *k* foreground scores. Finally, the two scores are spliced together to obtain the foreground-background probability score *S*_*fbg*_.

The original labels generated using the CAM after seed region refinement still lack detailed information. Therefore, in this paper, CRF is used to remove some of the mislabeled pixels and obtain CRF pseudo-labels possessing high-confidence foreground detail information, with the expression notated as:

$$L_{CRF}=CRF(I,[S_{fg},S_{bg}]),$$ (11)

Although the original label has a basically complete foreground region, many of the background pixels are classified as foreground pixels with a misclassification phenomenon, which disguises the fact that the background pixel classification in the original label is more reliable. Therefore, in this paper, we draw on the thresholding method to choose a reliable threshold *α* and generate the base pseudo-label with more high-confidence background information based on $M_{CAM}^{k}$:

$$L_{base}(i)=\{\begin{array}{c} \underset{k\in K}{\arg\;\max}(S_{fbg}^{k}(i)),\\ 255, \end{array}\begin{array}{c} if\;\underset{k\in K}{\max}(S_{fbg}^{k}(i))>\alpha \\ else \end{array},$$ (12)

Where, *K* represents the complete set of classes including the background, and 255 indicates that the labeling class is still uncertain. At each position index *i*, the value with the highest probability score in category *K* is selected and compared with the threshold *α*. If it is greater than *α*, the score is set as the base pseudo-label; otherwise, it is set as a white pixel, representing a low-confidence region.

The final input to the semantic segmentation branch, serving as the generative expression for pseudo-labels used in training, is as follows:

$$L_{final}(i)=\{\begin{array}{c} L_{base}(i),\\ 255, \end{array}\begin{array}{c} ifL_{base}(i)=L_{CRF}(i)\\ else \end{array},$$ (13)

Eq (13) can be understood as an operation that computes the intersection of the two pseudo-labels, leaving the non-overlapping regions as white pixels. This preserves both the foreground region, rich in boundary detail information from the CRF pseudo-label, and the reliable background region screened by the base pseudo-label, thereby enhancing the overall quality of the pseudo-label.

### 3.6 Loss function

The loss function for the entire network consists of three components: a cross-entropy loss function, an average absolute error loss function, and an energy loss function.

The cross-entropy loss function for classifiers is defined as follows:

$$L_{cls1}=-\frac{1}{K}\sum_{k=1}^{K}\left(y_{k}\log(\frac{1}{1+e^{-{\hat{y}}_{k}}}\right)+(1-y_{k})\;\log(1-\frac{1}{1+e^{-{\hat{y}}_{k}}})),$$ (14)

Where *y* is the image-level label and $\hat{y}$ is the prediction vector for image classification obtained after the final global average pooling layer of the classifier.

Similarly, we can get the loss function for other classifiers:

$$L_{cls2}=L_{1/2}+L_{1/4},$$ (15)

Where *L*_1/2_ and *L*_1/4_ denote the cross-entropy loss function obtained when the input image is split into 4 and 16 blocks, respectively, and propagated to the classifier.

In the Multilevel Activation Fusion module, the inputs are the merged CAM and the original CAM. While merged CAM captures a broader range of features, it may also recognize erroneous features, such as background elements, in each patch. Conversely, original CAM captures fewer features but tends to identify the most discriminative and accurate ones. To address this, we use the mean absolute error (MAE) as a constraint to narrow the gap between merged CAM and original CAM. This approach leverages the strengths of both CAMs to mitigate their weaknesses. By enhancing the quality of both CAMs, the Multilevel Activation Fusion module produces an improved enhanced CAM. The expression is as follows:

$$L_{SE}={\left\|M_{merge}-M_{MS}\right\|}_{1},$$ (16)

Where the *M*_*merge*_ is merged CAM obtained from Eq (5) and the *M*_*MS*_ is original CAM obtained from Eq (2).

In the Multilevel Activation Fusion module, the inputs are the merged CAM and the original CAM. While the merged CAM captures a broader range of features, it may also recognize erroneous features, such as background elements, in each patch. Conversely, the original CAM captures fewer features but tends to identify the most discriminative and accurate ones. To address this, we use the mean absolute error (MAE) as a constraint to narrow the gap between the merged CAM and the original CAM. This approach leverages the strengths of both CAMs to mitigate their weaknesses. By enhancing the quality of both CAMs, the Multilevel Activation Fusion module produces an improved enhanced CAM. The expression for this constraint is as follows:

$$L_{CE}=-\sum_{k\in K}B_{k}(i)\log(P_{net}^{k}(i)),$$ (17)

Where *B*_*k*_(*i*) is a binary number equal to 1 if the result of segmentation at position index *i* is category *k*, and 0 otherwise. $P_{net}^{k}(i)$ denotes the probability of category *k* output by the segmentation head at position index *i*.

As the pseudo-labels generated in part 3.5 exclusively designate high-confidence regions, the remaining unlabeled areas are assigned white pixels. To predict these unlabeled regions, we introduce an energy loss function that takes into account the RGB color and spatial location properties of the image. The expression for this loss function is as follows:

$$L_{energy}=\sum_{i=0}^{N}\sum_{\begin{array}{c} j=0\\ j\neq i \end{array}}^{N}E(i,j),$$ (18)

Where, *E*(*i*,*j*) denotes the energy equation between pixels *i* and *j* in a given image *I* [30]:

$$E(i,j)=\sum_{\begin{array}{c} k_{a},k_{b}\in K\\ k_{a}\neq k_{b} \end{array}}G(i,j)P_{net}^{k_{a}}(i)P_{net}^{k_{b}}(j),$$ (19)

Where *k*_*a*_ and *k*_*b*_ are the different category labels, $P_{net}^{k_{a}}(i)$ and $P_{net}^{k_{b}}(i)$ are the category probabilities output by the segmentation head at pixel *i* and *j*. *G*(*i*,*j*) is the Gaussian kernel function. It takes into account the characteristics of the image’s RGB color and spatial location to improve the classification accuracy of each pixel of pseudo-labels. Its expression is shown as follows:

$$G(i,j)=\frac{1}{W}\exp(-\frac{{\left\|I_{s}(i)-I_{s}(j)\right\|}^{2}}{2\sigma_{d}^{2}}-\frac{{\left\|I_{rgb}(i)-I_{rgb}(j)\right\|}^{2}}{2\sigma_{r}^{2}}),$$ (20)

Where 1/*W* is the normalization constant, *I*_*s*_(∙) is the pixel spatial location of the image, *I*_*rgb*_(∙) is the RGB color of the corresponding position, *σ*_*d*_ and *σ*_*r*_ are hyper-parameters controlling the Gaussian kernel scale. The Gaussian kernel energy loss function takes into account the characteristics of the image’s RGB color and spatial location to improve the classification accuracy of each pixel of pseudo-labels. Eq (19) is simplified using the Potts model [31]:

$$E(i,j)=G(i,j)\sum_{k\in K}P_{net}^{k}(i)(1-P_{net}^{k}(j)),$$ (21)

Ultimately, the loss function expression for the entire network can be written in the following form:

$$L_{loss}=L_{cls1}+L_{cls2}+L_{SE}+L_{CE}+L_{energy},$$ (22)

## 4. Experimental results and discussion

### 4.1 Data sets and implementation details

Our proposed model is first pre-trained based on Image Net dataset. It is then further trained on PASCAL VOC 2012 dataset and evaluated. The PASCAL VOC dataset consists of 20 foreground classes and 1 background class, further divided into three subsets: a training set comprising 1464 images, a validation set with 1449 images, and a test set comprising 1456 images. To augment the training set, we incorporate additional data from the literature [32], totaling 10582 images. In our experiments, we exclusively utilize image-level labels and employ Mean Intersection over Union (mIoU) as the metric to assess the effectiveness of our method on an image dataset with 21 categories.

Due to the large number of parameters in Transformer models, training typically requires significant computational resources and time, especially when handling large-scale semantic segmentation tasks. This can result in excessively long training times or necessitate expensive hardware support.Therefore, we chose a residual network with 38 convolutional layers [33] as the backbone network. This network eliminates all the original pooling layers, replaces the fully connected layers with global average pooling, and modifies the convolution in the last three residual cell blocks. The dilation rate is set to 2 for the third layer and 4 for the last two layers. In the energy loss function, the values of *σ*_*d*_ and *σ*_*r*_ are set to 100 and 15, respectively.

The training images were resized with a scale randomly sampled from (0.5, 1.5) and subjected to random flips. Subsequently, the images were normalized and randomly cropped to a size of 321×321. The batch size was set to 2, and the maximum number of iterations was 10,000. The multiscale coefficients in Eq (2) were set to *S*_*i*_ = {*S*_1_,*S*_2_,*S*_3_,*S*_4_} = {0.5,1,1.5,2}, *γ* in Eq (10) was set to 4, the CRF parameter in Eq (11) was set according to the literature [34], and *α* in Eq (12) was set to 0.5. During training, each branch updated the backbone network. During testing, only the split branch was used for prediction.

All experiments were conducted using PyCharm software within the PyTorch environment. Due to our limited hardware resources, the experimental results of all methods in this article were obtained through parameter training and calculation using the NVIDIA GTX 1060 graphics card.

### 4.2 Ablation experiments and methods analysis

In the subsequent experiments, we employed a baseline model without the Location Expansion Module, Multilevel Activation Fusion Module, and Foreground-background Enhanced Module. This baseline model is a simple two-branch segmentation network, consisting of a classification branch and a segmentation branch.

#### 4.2.1 Selection of the number of image blocks

**Fig 8:**
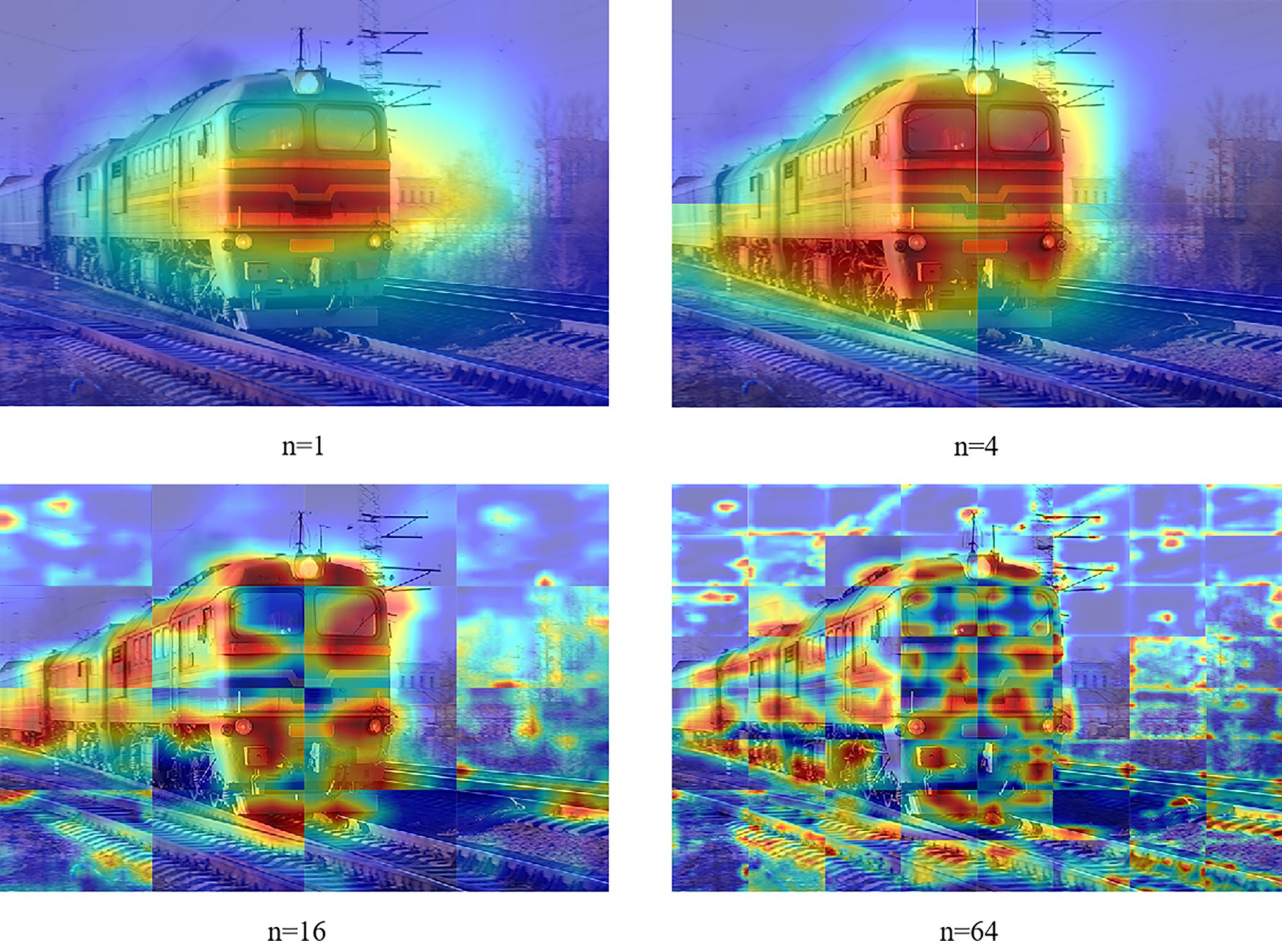
CAM with different number of split blocks on PASCAL VOC 2012 dataset. They represent the CAMs generated by the baseline model, the CAMs generated with *n* = 4, the CAMs generated with *n* = 16, and the CAMs generated with *n* = 64, respectively.2.

This section investigates the impact of the number of image splits (denoted as *n*) on the segmentation results within the Location Expansion Module. As depicted in Table 1, there is a noticeable improvement in mIoU as *n* increases. However, a decrease in mIoU is observed when *n*=64. The reason for this is that as *n* increases, objects are overrepresented in blocks, resulting in a weakening of the classification network’s ability to capture features based on context. Furthermore, the proportion of non-relevant features in the blocks increases. Consequently, the classifier will be forced to identify the wrong features in the blocks that otherwise have no relevant features. As illustrated in the 2nd and 3rd images of Fig 8 , as *n* increases, the body features covered by CAM are gradually sparse and discontinuous. Furthermore, the classifier will erroneously identify additional features (e.g., tracks, sky, trees). Conversely, as illustrated in the 2nd image, when *n* is reduced, the classifier’s ability to identify features is enhanced, resulting in a more continuous and comprehensive feature area.

**Table 1:** Ablation experiments for the number of image blocks in location expansion module.

*n*=4	*n*=16	*n*=64	mIoU (%)
√			51.24
	√		52.03
		√	50.76
√	√		53.82
√		√	52.62
	√	√	51.58
√	√	√	**53.91**

Additionally, the classifier’s ability to accurately recognize small details is attributed to the over-representation of features due to an excessively large *n*. As illustrated in the 2nd image the CAM exhibits limited coverage in the rear of the carriages and the bottom of the locomotive. However, in the 3rd and 4th images, these fine features are more comprehensively captured. Consequently, we explored the potential of cascading different CAM. When cascading the aforementioned three CAMs, the mIoU reaches up to 53.91%. However, when compared to cascading *n*=4 and *n*=16 CAM, the mIoU is only improved by 0.09%. Furthermore, cascading the three further increases the computational cost. Therefore, we have chosen the scheme of cascading *n*=4 and *n*=16 to expand the seed region.

#### 4.2.2 Selection of the number of feature fusion layers

The ResNet38 network comprises seven stages of convolutional blocks. Jiang [15] suggested that fusing multi-scale features at various levels can enhance the self-attention mechanism’s capability to capture both detailed information and global context, thereby improving the model’s perceptual ability and performance. Based on this principle, in this section, we investigate the effect of different stages of feature maps at various scales, connected by up-sampling, on the segmentation results. CAM is extracted stage by stage based on the feature map, allowing for a detailed analysis of its contribution to the segmentation process.

**Fig 9:**
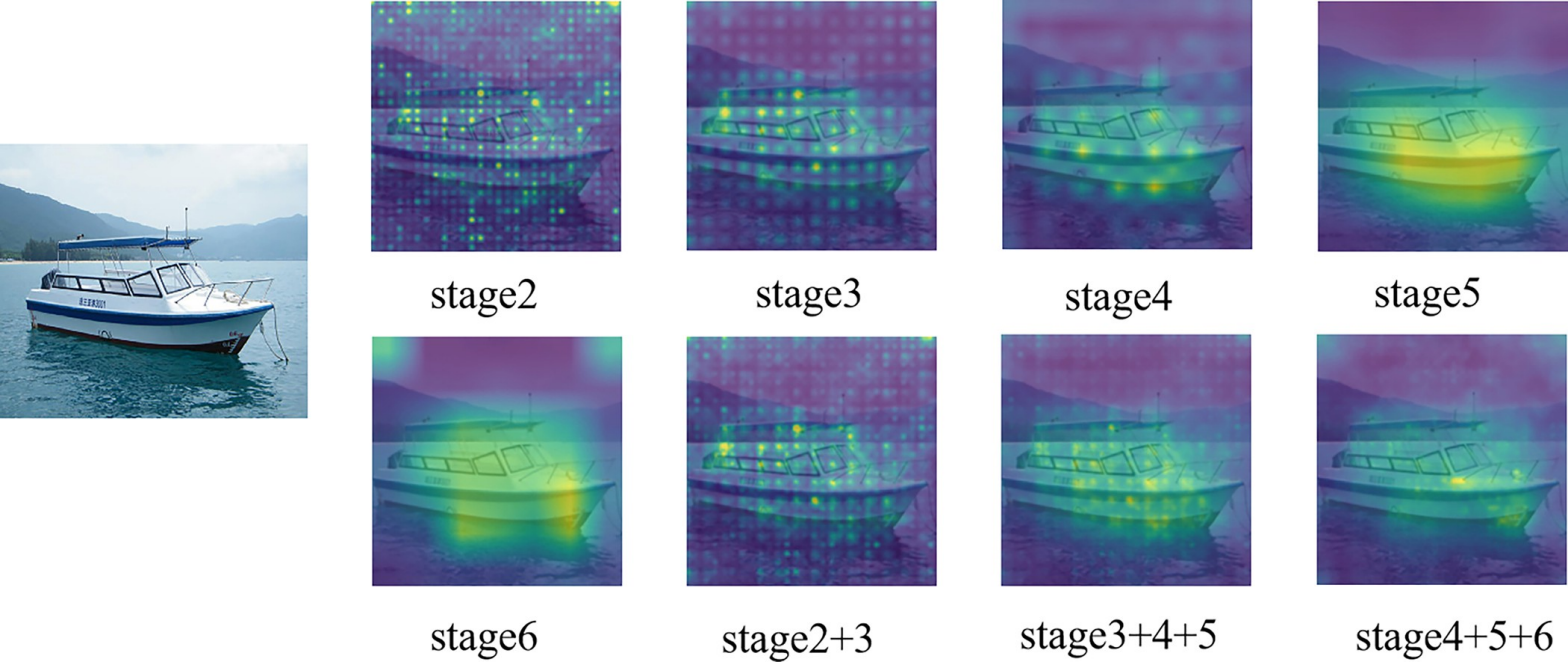
CAM at different stages on PASCAL VOC 2012 dataset. The images in Fig 9 are, in order: Input image; CAMs extracted after the second to sixth stage; CAM fused in 2nd and 3rd stage; CAM fused in2nd, 3rd, and 4th stage; CAM fused in 3rd, 4th, and 5th stage; CAM fused in 4th, 5th, and 6th stage.

As depicted in Table 2, the mIoU increases as the stage of the input feature map of the Multilevel Activation Fusion Module deepens. Because as the convolutional layers deepen and the sensory fields keep stacking up, the network acquires more complete semantic information, and the CAM covers denser and more complete targets, as shown in the first five CAMs in Fig 9. However, the image information is compressed and the network is less able to perceive its details.

**Table 2:** Ablation experiments for integrating feature maps from different stages.

Stage=1	Stage=2	Stage=3	Stage=4	Stage=5	Stage=6	Stage=7	mIoU (%)
√							41.42
	√						42.36
		√					43.21
			√				47.55
				√			49.81
					√		50.23
						√	50.94
					√	√	51.08
				√	√		53.53
			√	√	√		54.61
		√	√	√			**55.82**
	√	√	√	√			55.36
√	√	√	√	√			54.74

Conversely, shallow features inherently contain more fine-grained location information. Thus, we propose to fuse both shallow and deep features. As depicted in the last three CAMs in Fig 9, the superposition of stages enables the feature map to incorporate detailed location information while retaining complete semantic information. Notably, the feature maps fused in the third, fourth, and fifth stages exhibit the most significant improvement in mIoU. However, mIoU decreases after fusing the feature maps of the sixth stage. This decline can be attributed to deeper features potentially capturing irrelevant features, such as the sky depicted at the top in the fifth CAM in Fig 9. Furthermore, continuing to fuse shallower features beyond the third, fourth, and fifth stages also results in a decrease in mIoU. This is due to shallower features lacking high-level semantic information and containing noise, which adversely affects the efficacy of feature fusion. From these observations, it is evident that the optimal feature layer for fusion should strike a balance between being neither too shallow nor too deep.

#### 4.2.3 Selection of pseudo label thresholds

**Fig 10:**
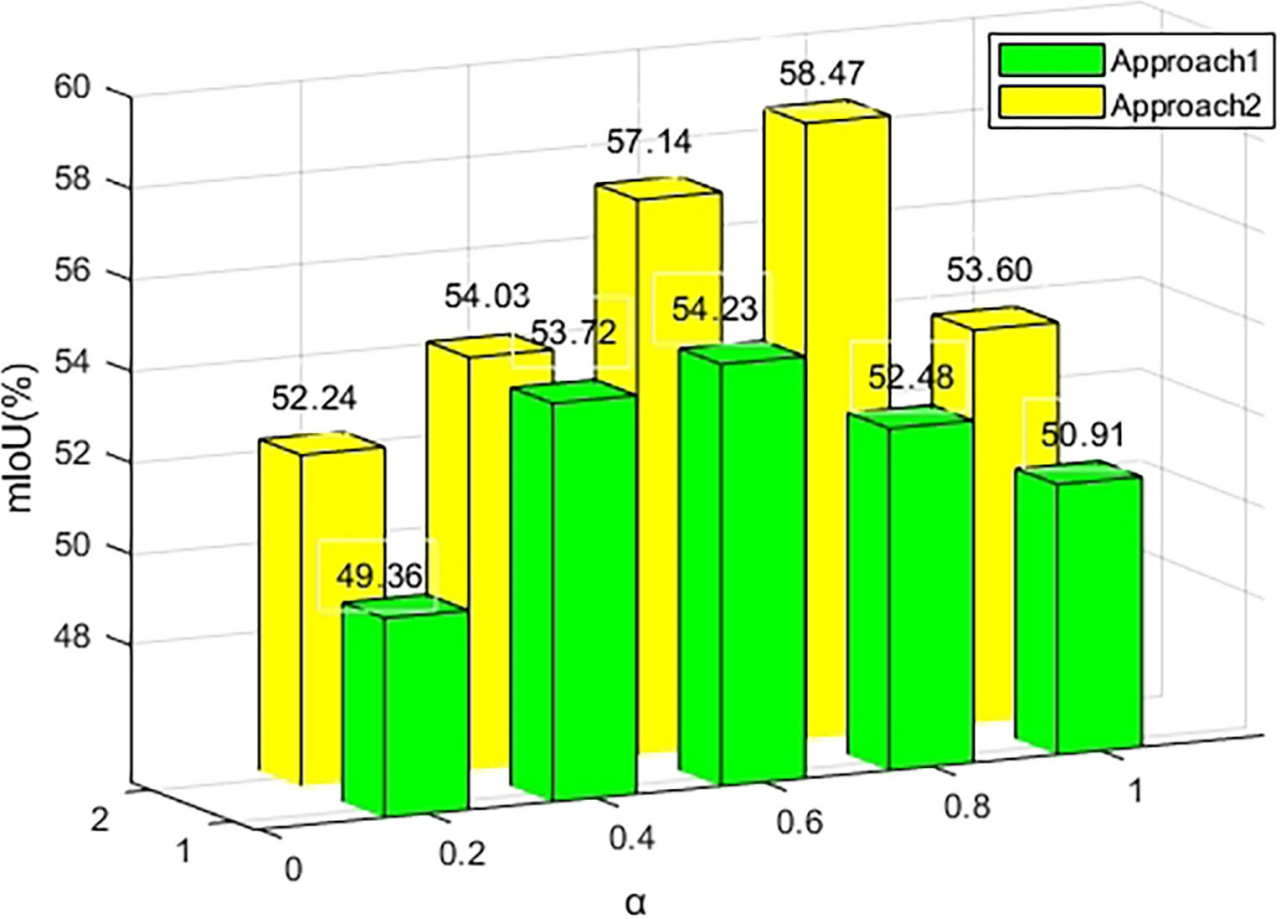
Results of different thresholds on segmentation results. When *α*=0.6, the maximum value of mIoU in Approach 1 is 54.23%; When *α*=0.8 in Approach 2, the maximum mIoU is 58.47%.

This section investigates the impact of different thresholds, denoted as *α*, on segmentation results when generating base pseudo-labels. *α* smaller A indicates retaining reliable and numerous pixel information, while a larger *α* retains more reliable but less numerous pixel information. In this experiment, mIoU values for the semantic segmentation network were calculated for two scenarios: the base model combined with the Foreground-background Enhancement Module (Approach 1) and the base model combined with the Location Expansion Module, Multi-level Fusion Activation, and Foreground-background Enhancement Module (Approach 2). As depicted in Fig 10, Approach 2, incorporating localization expansion and multilevel fusion activation modules, consistently outperforms Approach 1 for any value of *α*. Optimal mIoU scores of 54.23% and 58.47% are achieved at *α* = 0.6 and *α* = 0.8, respectively. The following conclusions are drawn: First, a smaller *α* = 0.6 yields more labeled pixels, but introduces misclassifications, adding errors to the model. Second, a larger *α* provides higher-confidence labeled pixels, but the number of such pixels is small, limiting the network’s training. Third, as Approach 2 enhances CAM’s quality with the localization extension and multilevel fusion activation module, it obtains more high-confidence pixels when generating pseudo-labels. Therefore, as *α* increases, Approach 2 acquires more reliable labeling information, further improving segmentation effectiveness.

#### 4.2.4 Ablation experiments with improved modules

The experiments conducted in this section showcase the results obtained by incrementally adding the modules designed in this paper to the baseline. As shown in Table 3, adding a single module or cascading multiple modules consistently enhances segmentation performance. When all three modules are incorporated simultaneously, there is a notable improvement in mIoU, with an increase of 7.62% over the original baseline.

**Table 3:** Ablation experiments with three modules combinations.

Baseline	Location Expansion Module	Multilevel Activation Fusion Module	Foreground-background Enhanced Module	mIoU(%)
√				50.85
√	√			53.82
√		√		55.79
√			√	54.23
√	√	√		56.76
√	√	√	√	**58.47**

**Fig 11:**
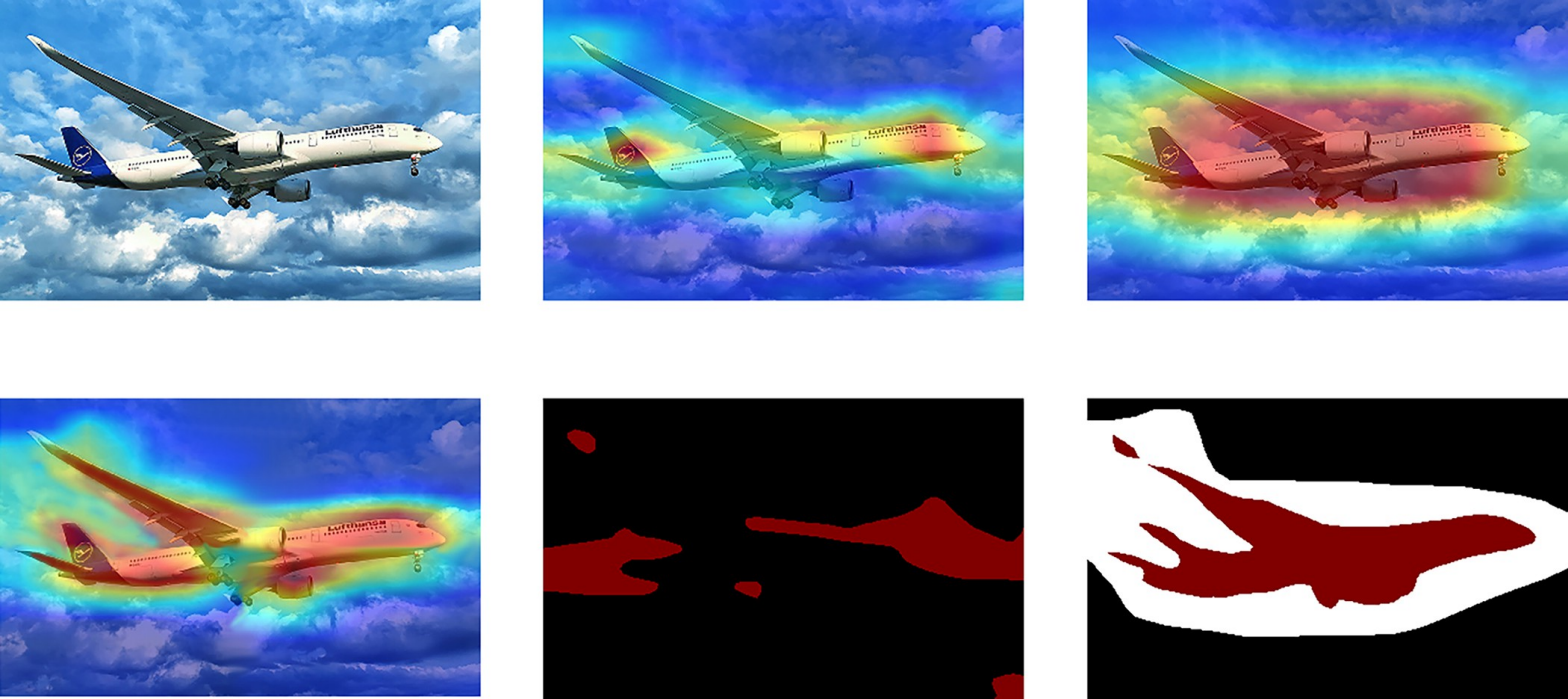
Visualization results of ablation experiments with three module combinations on PASCAL VOC 2012 dataset. The images in Fig 11 are, in order: Input picture; CAM generated by baseline; Generated by baseline + Location Expansion Module; Generated by baseline + Location Expansion Module + Multilevel Activation Fusion Module; Pseudo-label generated by the base line and generated by baseline+Location Expansion Module + Multilevel Activation Fusion Module + Foreground-background Enhanced Module.

As shown in the 2d image in Fig 11 the CAM of the baseline illustrates the classifier’s focus on high discriminative parts such as the head and tail of the airplane. Subsequently, the 3rd image demonstrates the CAM after integrating the Location Expansion Module. Here, the classifier encompasses nearly all aircraft features; however, this over-extension may result in certain background pixels receiving undue attention, potentially leading to misclassification. Refinement and boundary convergence are evident in the 4th image following the addition of the Multilevel Activation Fusion Module. The 5th image showcases the pseudo-label generated by the base model, displaying ineffective segmentation. By incorporating the Foreground-background Enhanced Module based on the first two modules, as depicted in the last image, the pseudo-labeling process adopts a different approach. Rather than forcing pixels with low confidence scores near the threshold into foreground or background categories, uncertain categories are denoted by white labels. This approach yields pseudo-labels closer to reality, as it only assigns labels to pixels with high confidence and rich detail.

#### 4.2.5 Comparison with existing methods

To assess the performance of the network we proposed in this paper, this section compares the OSTE model with two other end-to-end supervision semantic segmentation algorithms, namely EA-Adapt [35] and RRM [36]. As presented in Table 4, the OSTE network, utilizing the threshold selection method, effectively identifies higher-confidence background pixels as pseudo tags. Consequently, the background segmentation accuracy improves by 13.36% and 3.89% compared to EA-Adapt and RRM, respectively. Across other categories, the OSTE network consistently achieves higher MIOU scores. Notably, for intricate boundary information, such as plants, the OSTE network demonstrates a substantial increase of 16.51% and 13.78% compared to EA-Adapt and RRM, respectively. By introducing CRF to obtain a pseudo-label with high settlement prospects, the OSTE significantly enhances segmentation accuracy, particularly for challenging prospects.

**Table 4:** Comparison of mIoU with EM-Adapt and RRM based on VOC2012 validation set.

**(a) is IoU for categories 1 to 11 corresponding to three methods.**																						
		background		plane		bike		bird		boat		bottle		bus		car		cat		chair		cow
EM-Adapt		74.84		35.62		20.44		40.15		24.65		37.03		49.36		43.22		43.48		15.21		39.37
RRM		84.31		61.16		27.94		63.40		29.51		53.85		68.34		65.97		66.04		26.17		51.14
Ours-OSTE		88.20		62.63		31.97		67.77		42.39		59.55		72.56		73.08		74.95		32.26		58.02
**(b) is IoU for categories 12 to 21 and mIoU corresponding to three methods.**																						
	table		dog		horse		mbk		person		plant		sheep		sofa		train		monitor		mIoU(%)	
EM-Adapt	28.98		47.62		46.82		55.11		28.73		31.52		45.03		30.29		35.06		47.91		39.07	
RRM	40.65		60.93		56.26		64.08		66.96		34.25		61.89		33.12		48.54		57.77		53.44	
Ours-OSTE	37.79		65.08		59.93		66.63		71.12		48.03		64.55		39.59		58.67		53.03		**58.47**	

In this paper, we compare the OSTE model with semantic segmentation algorithms in recent years to further validate the effectiveness of the method in this paper. In the types of supervision , F denotes full supervision and W-P denotes weak supervision based on image labeling. As can be seen from Table 5, although there is still a large gap between weak supervision and full supervision, the segmentation performance of the OSTE has also equaled or even surpassed that of the classical FCN semantic segmentation network. ISISU and Fickle-Net achieve high mIoU, but both of them use the saliency detection dataset MARA-B as additional supervisory information and are trained with additional networks respectively. OSTE also surpasses some weakly supervised methods that do not require additional data as supervisory information.

**Table 5:** Comparison of the segmentation results based on three methods under the VOC2012 validation set.

Method	Backbone	Types of supervision	One step	Extra data	mIoU(val)
FCN [37]	VGG-16	F	No	-	58.16
Deeplab-v2 [38]	Resnet-101	F	No	-	70.12
ISISU [39]	Resnet-101	W-P	No	MSRA-B	56.43
Fickle-Net [40]	Resnet-101	W-P	No	MSRA-B	57.81
Affinity-Net [17]	Resnet-38	W-P	No	-	51.68
IRN [16]	Resnet-50	W-P	No	-	54.41
SEAM [23]	Resnet-38	W-P	No	-	55.59
ECS-Net [41]	Resnet-38	W-P	No	-	58.64
CG-Net [42]	Resnet-101	W-P	No	-	59.75
EM-Adapt [35]	VGG-16	W-P	Yes	-	39.07
RRM [36]	Resnet-38	W-P	Yes	-	53.44
Ours-OSTE	Resnet-38	W-P	Yes	-	**58.47**

However, the mIoU of ECS-NET and CG-NET is slightly higher than OSTE There may be the following reasons:

Firstly, ECS-NET uses more attention modules than OSTE to enhance the feature localization ability of CAM. Secondly, ECS-NET applies iterative erasure and suppresses erasure noise. But the end-to-end approach, causes noise to propagate into the segmentation network, which may cause the network to learn inaccurate information during training. And then, due to GPU limitation, the residual network of OSTE has only 38 layers, but its mIoU metric closely rivals that of CG-NET, which employs a 101-layers residual network. Finally, neither of these networks adopts an end-to-end structure. The researcher aims to optimize the generation of both pseudo-labels and semantic segmentation models, potentially resulting in enhanced performance but with a significant increase in computational demands. In contrast, OSTE can simultaneously generate pseudo-labels and train segmentation networks, thereby reducing model complexity. Notably, OSTE achieves an mIoU that surpasses many complex two-step models and approaches the performance of the aforementioned superior two-step models.

Comparing to the weakly supervised approach that also uses the end-to-end structure, the performance of OSTE is improved by 19.4% and 5.03% compared to EM-Adapt and RRM, respectively.

**Fig 12:**
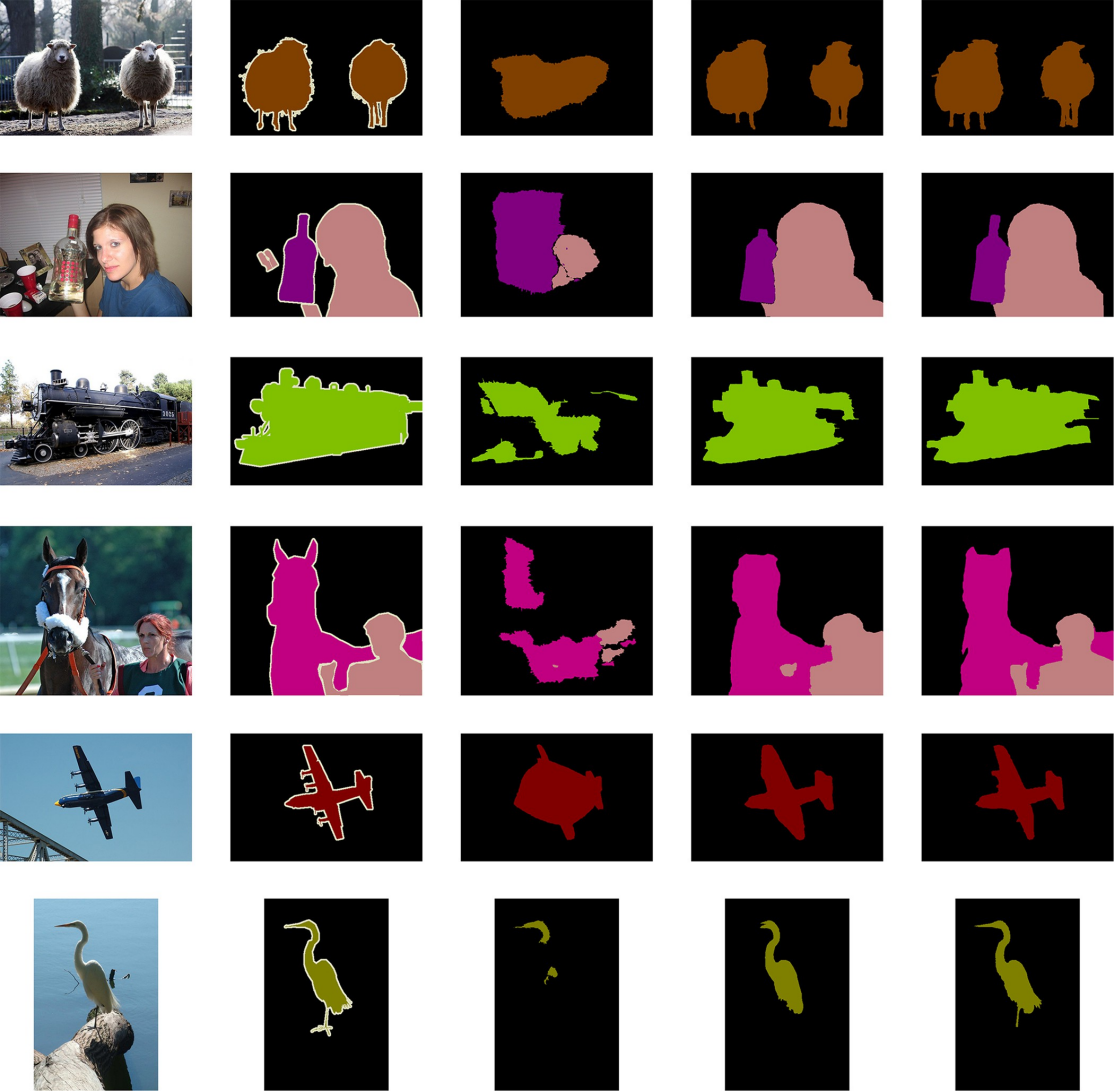
Qualitative segmentation results on the PASCAL VOC 2012 validation set. The first column represents origin image, the second column represents ground truth, the third column represents the results of EM-Adapt, the fourth column represents the result of RRM, and the fifth column represents ours result.

To demonstrate the segmentation results more intuitively, OSTE qualitatively compares the segmentation results with the end-to-end methods EM-Adapt and RRM based on the PASCAL VOC 2012 validation set. As shown in Fig 12: the OSTE has a broader semantic annotation for large objects, such as train bodies; while the segmented boundaries of fine objects are more accurate and smooth, such as cat’s ears, sheep’s legs, legs, airplane wings, legs of chair, and bird legs. Among these three methods, the OSTE in this paper has the best segmentation performance.

## 5. Conclusion

In this paper, for the existing weakly-supervised semantic segmentation models with cumbersome training steps, complex network structure and poor segmentation performance, we propose an end-to-end weakly-supervised semantic segmentation network OSTE based on image-level labels and experimentally validate it on the PASCAL VOC 2012 dataset, and draw the following conclusions.

First, to enhance the acquisition of image location information and extend seed areas, we introduce a Location Expansion Module. It compels the classifier to look beyond the most distinctive parts of the object, enabling CAM to combine local information from the image and cover a more comprehensive semantic region. The network achieves a balance between complexity and segmentation performance when the original image is divided into 4 and 16 segments of CAM. This results in a 2.97% increase in MIOU over the basic model, reaching 50.85%.

Second, in terms of refining the seed areas, we design a Multilevel Activation Fusion Module, which uses the pixel correlation of multilevel fusion features to guide the CAM to obtain more global contextual information and improve the prediction of the CAM at the object boundary. When fusing the third, fourth, and fifth layers of Resnet38 features, it has more accurate inter-pixel correlations with CAM, and the mIoU is improved by 4.94% compared to the base model.

Third, in the context of pseudo-label generation, we introduce a Prospective-background Enhanced Module. Leveraging CRF, it generates a foreground label with reliable details and screens a trustworthy background label using threshold *α*. These are then combined to form a pseudo-label with improved quality. This approach results in a 3.38% increase in MIOU compared to the basic model.

Fourth, after adding the above three modules at the same time, mIoU is improved by 7.62% compared to the original base model. In comparison with related semantic segmentation methods, this paper’s method improves 0.31% compared to the most classical fully supervised segmentation network FCN; it improves 19.4% and 5.03% compared to EM-Adapt and RRM, which are also end-to-end one-step trainings, and has a clear advantage; compared with the two-step weakly supervised method, which is more cumbersome to train and yet has a higher accuracy, this paper’s method still outperforms and has come close to the performance of the original base model in segmentation. Compared with the current two-step weakly supervised method, which is more tedious to train but has higher accuracy, the method in this paper still surpasses and is close to the ECS-Net and CG-Net, which have more advanced performance.

Starting from the CAM graph and pseudo-labeling in the classification branch, this method can be used in the future for additional innovative research in the segmentation branch to completely surpass the two-step weakly-supervised method and continue to approach the segmentation performance of the fully-supervised method.
